# Does the Type of Permanent Mesh Matter for Inguinal Hernia Repair?

**DOI:** 10.1007/s10029-026-03615-9

**Published:** 2026-04-28

**Authors:** Nandita Nettu Mahajan, Victor Heh, Nicole Buchely, Prashanth Sreeramoju

**Affiliations:** 1 Department of Surgery, Montefiore Medical Center, The University Hospital for Albert Einstein College of Medicine, 1250 Waters Place, Hutch Tower II, 11th floor, Bronx, NY 10461 USA; 2 Department of Surgery, The Ohio State University Wexner Medical Center, Grandview Heights, OH USA

**Keywords:** Inguinal hernia, Inguinal hernia repair, Mesh repair, Polyester mesh, Polypropylene mesh, Groin hernia repair

## Abstract

**Introduction:**

Inguinal hernia repair (IHR) is one of the most performed general surgery procedures in the United States, with more than 700,000 cases annually. There are limited data on the clinical outcomes based on the type of mesh (polyester or polypropylene) used in IHR. This study aims to bridge the knowledge gap on clinical outcomes for polyester and polypropylene mesh used for open and minimally invasive IHR.

**Patients and methods:**

A retrospective review of prospectively collected data from Abdominal Core Health Quality Collaborative (ACHQC) of all adult patients (Age ≥ 18 and ≤ 90 years) who underwent initial elective IHR (2014–2024) with or without mesh. Univariate and multivariate analyses were performed comparing mesh-based repair with no-mesh repair as the control group.

**Results:**

From 37,262 patients with initial elective IHR, 25,331 had polypropylene mesh, 8391 had polyester mesh, and 1770 had no-mesh repair. At 30-day follow-up, polypropylene and polyester had lower readmission (0.8% and 0.7% vs 1.3%;*p* < 0.05) but higher surgical site occurrences (SSO) (5.3% and 5.5% vs 2.2%;*p* < 0.05) compared to no-mesh repair. At 1-year follow-up, polypropylene and polyester had lower recurrence compared to no-mesh repair (6.4% and 6.6% vs 11%; p < 0.05). Additional analyses demonstrated similarly lower recurrence with polyester mesh. On logistic regression, polyester (OR 0.4,CI 0.28–0.69) and polypropylene (OR 0.4,CI 0.27–0.57) were protective against recurrence with similar SSO for polyester (OR 1.9,CI 1.01–3.51) and polypropylene (OR 1.9,CI 1.06–3.51) compared to no-mesh repair. Polyester (OR 0.4,CI 0.31–0.56) and polypropylene (OR 0.5,CI 0.39–0.65) had lower ≥6-month EuraHS-QoL pain score compared to no-mesh repair. No statistically significant difference for polyester versus polypropylene based on surgical approach.

**Conclusion:**

There was no difference in the 30-day SSO or 1-year hernia recurrence for polyester and polypropylene, irrespective of the surgical approach. Contrary to generalized belief, synthetic mesh-based posterior approach repair may be protective against chronic pain. This highlights the need to focus on surgeon preference and resource utilization that could impact practice guidelines.

**Graphical Abstract:**

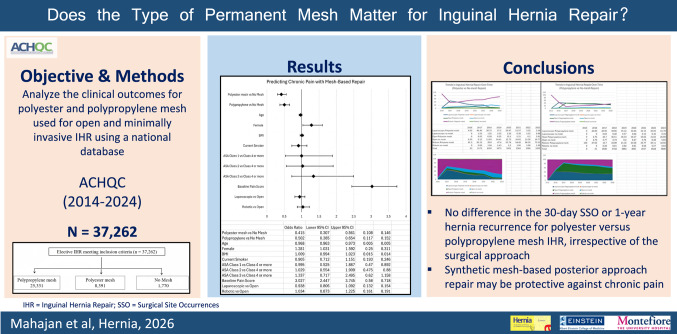

**Supplementary Information:**

The online version contains supplementary material available at 10.1007/s10029-026-03615-9.

## Introduction

Inguinal hernia repair (IHR) is among the most commonly performed general surgery procedures in the United States, with more than 700,000 cases annually [1, 2]. International guidelines recommend mesh reinforcement for all elective IHR to decrease recurrence, which is the standard of care irrespective of open versus minimally invasive approach for IHR [3, 4]. However, the literature suggests that mesh repairs might have been associated with chronic pain and daily lifestyle limitations [5, 6]. In addition, the surgical approach and mesh selection are tailored based on patient characteristics, resource availability, and surgeon expertise, leading to worldwide and regional variations in practice [7].

Over the last decade, there has been considerable advancement with the development of a variety of permanent mesh focusing on porosity, polymer weight, foreign body reaction, anatomic compliance and biologic integration [7, 8]. Limited randomized controlled studies by Prakash et al. and Kenary et al. report no significant differences in the overall outcome for light weight versus heavy weight mesh with comparable quality of life (QoL) for laparoscopic and open IHR [8, 9]. However, these two studies were conducted in developing nations with different patient populations, limiting generalization within the United States.

A Sweden national registry-based study by Melkemichel et al. demonstrated increased recurrence risk with lightweight open anterior IHR while meta-analysis by Wu et al. demonstrated similar outcome of increased recurrence with lightweight mesh for laparoscopic IHR [10, 11]. While a more recent retrospective cohort study by Calomino et al. reported reduced discomfort from lightweight mesh repair without increased in recurrence risk for open IHR [12, 13].

While lightweight and heavyweight designations reflect mesh density, emerging literature highlights material type as an important determinant of patient outcomes, underscoring the need to better delineate differences between polypropylene and polyester meshes. Recent studies examining different mesh materials in IHR reflect this shift in focus, but report heterogeneous findings across postoperative pain, seroma formation, and functional recovery [13–17]. This highlights the need to understand better and define the clinical outcomes of different mesh material types used for open and minimally invasive IHR. This study aims to bridge that knowledge gap by analyzing the clinical outcomes for polyester and polypropylene mesh used for open and minimally invasive IHR using a national database.

## Patients and methods

Abdominal Core Health Quality Collaborative (ACHQC) is a validated nationwide registry containing clinical, demographic, operative, and postoperative variables, including preoperative, intraoperative, 30-day, and 1-year postoperative data from institutions in the United States. We performed a retrospective review of prospectively collected data from ACHQC of all adult patients (age ≥ 18 and ≤ 90 years), who underwent initial elective open, laparoscopic, or robotic IHR from 2014 to 2024 with or without mesh.

Once patients were identified, we queried ACHQC for each patient’s information to create a retrospective database. Data collected included demographics (age, gender, race, co-morbidities, body mass index, ASA), operative information (date of surgery, type of surgery, operative time, type of mesh), and 30-day outcomes of Surgical Site Infection (SSI), Surgical Site Occurrences (SSO), Surgical Site Occurrence Requiring Surgical Intervention (SSOSI), readmission, European Hernia Society (EuraHS) QoL pain domain score and 1-year hernia recurrence. One-year hernia recurrence was defined using the ACHQC composite recurrence variable, which incorporates both patient-reported and surgeon-reported recurrence within one year. Chronic pain was defined as persistent pain for 6 months or more after the date of surgery. Patients with recurrent inguinal hernias, mesh removal procedures, concomitant repair of other abdominal wall defects such as ventral hernias, flank and lumbar hernias, neurectomies, and use of mesh other than polyester or polypropylene were excluded.

All the categorical variables were expressed as frequencies (n) with percentages and continuous variables as medians with interquartile range (IQR). Univariate and multivariate analyses were performed comparing mesh-based repair with no-mesh repair as the control group. A multivariable logistic regression analysis was used to evaluate clinical outcomes for polyester versus propylene mesh versus no-mesh IHR. *P*-values < 0.05 were considered significant. Logistic regression analysis results were reported as adjusted odds ratios (aOR), 95% confidence interval (CI), and *p*-value. All statistical analyses were performed using the Statistical Analysis System (SAS) v9.4. An Institutional Review Board waiver was obtained.

## Results

We identified 37,262 patients who underwent initial elective IHR that met the inclusion criteria. Of these, 25,331 patients underwent IHR with polypropylene mesh, 8391 patients with polyester mesh, and 1770 patients underwent a no-mesh repair. At the 30-day follow-up, polypropylene and polyester mesh repair had lower readmission rate (0.8% and 0.7% vs, 1.3%; *p* < 0.05) but higher SSO (5.3% and 5.5% vs, 2.2%; p < 0.05) compared to no-mesh repair. At 1-year follow-up, polypropylene and polyester mesh repair had lower hernia recurrence compared to no-mesh repair (6.4% and 6.6% vs 11%; p < 0.05). On univariate analysis of patients with complete 1-year follow-up and mesh classification, polyester mesh repair was associated with a significantly lower 1-year hernia recurrence compared to no-mesh repair (1.9% vs 4.2%, *p* < 0.0001). This finding further supports the protective effect of permanent synthetic mesh in elective inguinal hernia repair. There were no statistically significant differences in recurrence outcomes between polyester and polypropylene mesh when stratified by surgical approach. Univariate analyses for polyester versus polypropylene versus no-mesh IHR are detailed in Tables 1 and 2. Detailed univariate recurrence comparisons are shown in Supplementary Table S1.

**Table 1:** Polyester versus No-Mesh IHR

Variable	Overall (*N* = 10,161)	No mesh (*N* = 1770)	Polyester (*N* = 8391)	P-Value
Age	60 (48, 70)	61.0 (48.0, 70.0)	60.0 (48.0, 70.0)	0.1577
Gender				< 0.0001
Female	1048 (10.3%)	286 (16.2%)	762 (9.1%)	
Male	9113 (89.7%)	1484 (83.8%)	7629 (90.9%)	
BMI greater than 40				0.0156
Yes	128 (1.3%)	12 (0.7%)	116 (1.4%)	
No	9985 (98.7%)	1751 (99.3%)	8234 (98.6%)	
Current Smoker				< 0.0001
Yes	1240 (12.2%)	131 (7.4%)	1109 (13.2%)	
No	8921 (87.8%)	1639 (92.6%)	7282 (86.8%)	
Smoker within one year				< 0.0001
Yes	1306 (12.9%)	141 (8.0%)	1165 (13.9%)	
No	8853 (87.1%)	1629 (92.0%)	7224 (86.1%)	
ASA Class				< 0.0001
ASA 1	1668 (16.6%)	290 (16.9%)	1378 (16.5%)	
ASA 2	5750 (57.2%)	1123 (65.3%)	4627 (55.5%)	
ASA 3	2502 (24.9%)	292 (17.0%)	2210 (26.5%)	
ASA ≥ 4	140 (1.4%)	14 (0.8%)	126 (1.5%)	
Baseline Pain				
At Rest	1 (0, 3)	1 (0, 4)	1 (0, 3)	0.0003
During Activities	3 (1, 6)	4 (1, 7)	3 (1, 6)	<.0001
EuraHS QoL Pain Domain Score				
At Baseline	8 (3, 15)	10.0 (3.0, 17.0)	8 (3, 15)	< 0.0001
At ≥6 months	0 (0, 2)	0 (0, 4)	0 (0, 2)	< 0.0001
EuraHS overall QoL score				
At Baseline	30 (14, 46)	34.0 (15.0, 53.0)	29.0 (14.0, 45.0)	< 0.0001
At ≥6 months	2 (0, 8)	3 (0, 10)	2 (0, 7)	0.008
At 6 months or more				
Pain at Rest	0 (0, 0)	0 (0, 0)	0 (0, 0)	0.0575
Pain during Activities	0 (0, 1)	0 (0, 2)	0 (0, 1)	0.0013
Operative approach				< 0.0001
Open	2187 (21.5%)	1500 (84.7%)	687 (8.2%)	
Laparoscopic	2384 (23.5%)	116 (6.6%)	2268 (27.0%)	
Robotic	5590 (55.0%)	154 (8.7%)	5436 (64.8%)	
Year of surgery				< 0.0001
2016	23 (0.2%)	1 (0.1%)	22 (0.3%)	
2017	1173 (11.5%)	133 (7.5%)	1040 (12.4%)	
2018	1183 (11.6%)	181 (10.2%)	1002 (11.9%)	
2019	1673 (16.5%)	287 (16.2%)	1386 (16.5%)	
2020	1831 (18.0%)	278 (15.7%)	1553 (18.5%)	
2021	1562 (15.4%)	292 (16.5%)	1270 (15.1%)	
2022	1386 (13.6%)	337 (19.0%)	1049 (12.5%)	
2023	1330 (13.1%)	261 (14.7%)	1069 (12.7%)	
Hernia Recurrence at 30-day Follow-up				0.7129
Yes	13 (0.2%)	3 (0.2%)	10 (0.1%)	
No	8473 (99.8%)	1496 (99.8%)	6977 (99.9%)	
Hernia Recurrence at 1-year on PRO/Surgeon Report				0.0002
Yes	231 (7.6%)	75 (11.0%)	156 (6.6%)	
No	2803 (92.4%)	607 (89.0%)	2196 (93.4%)	
Surgical Site Occurrences, 30-day Follow-up				< 0.0001
Yes	419 (4.9%)	33 (2.2%)	386 (5.5%)	
No	8067 (95.1%)	1466 (97.8%)	6601 (94.5%)	
Surgical Site Infection, 30-day Follow-up				0.0454
Yes	12 (0.1%)	5 (0.3%)	7 (0.1%)	
No	8474 (99.9%)	1494 (99.7%)	6980 (99.9%)	
Readmission, 30-day Follow-up				0.0141
Yes	65 (0.8%)	19 (1.3%)	46 (0.7%)	
No	8421 (99.2%)	1480 (98.7%)	6941 (99.3%)

**Table 2:** Polypropylene versus No-Mesh IHR

Variable	Overall (*N *= 27101)	No mesh (*N *= 1770)	Polypropylene (*N *= 25331)	P-Value
Age	62 (51, 71)	61.0 (48.0, 70.0)	62.0 (51.0, 71.0)	0.0007
Gender				<0.0001
Female	2373 (8.8%)	286 (16.2%)	2087 (8.2%)	
Male	24728 (91.2%)	1484 (83.8%)	23244 (91.8%)	
BMI ≥ 40				0.0634
Yes	305 (1.1%)	12 (0.7%)	293 (1.2%)	
No	26616 (98.9%)	1751 (99.3%)	24865 (98.8%)	
Current Smoker				0.0001
Yes	2733 (10.1%)	131 (7.4%)	2602 (10.3%)	
No	24368 (89.9%)	1639 (92.6%)	22729 (89.7%)	
Smoker within one year				<0.0001
Yes	2994 (11.1%)	141 (8.0%)	2853 (11.3%)	
No	24099 (88.9%)	1629 (92.0%)	22470 (88.7%)	
ASA Class				<0.0001
ASA 1	4535 (16.9%)	290 (16.9%)	4245 (16.9%)	
ASA 2	15190 (56.7%)	1123 (65.3%)	14067 (56.1%)	
ASA 3	6647 (24.8%)	292 (17.0%)	6355 (25.4%)	
ASA ≥ 4	407 (1.5%)	14 (0.8%)	393 (1.6%)	
Baseline Pain				
At Rest	1 (0, 2)	1 (0, 4)	1 (0, 2)	<0.0001
During Activities	3 (1, 6)	4 (1, 7)	3 (1, 6)	<0.0001
At 6 months or more				
Pain at Rest	0 (0, 0)	0 (0, 0)	0 (0, 0)	0.0007
Pain during Activities	0 (0, 1)	0 (0, 2)	0 (0, 1)	<0.0001
EuraHS QoL Pain Domain Score				
At Baseline	7 (3, 14)	10.0 (3.0, 17.0)	7 (3, 13)	<0.0001
At ≥ 6 months	0 (0, 2)	0 (0, 4)	0 (0, 2)	<0.0001
EuraHS overall QoL Score				
At Baseline	27 (13, 45)	34.0 (15.0, 53.0)	27.0 (13.0, 45.0)	<0.0001
At ≥ 6 months	2 (0, 6)	3 (0, 10)	1 (0, 6)	<0.0001
Operative approach				<0.0001
Open	10840 (40.0%)	1500 (84.7%)	9340 (36.9%)	
Laparoscopic	9430 (34.8%)	116 (6.6%)	9314 (36.8%)	
Robotic	6831 (25.2%)	154 (8.7%)	6677 (26.4%)	
Year of surgery				<0.0001
2015	4 (0.0%)	0 (0.00%)	4 (0.0%)	
2016	21 (0.1%)	1 (0.1%)	20 (0.1%)	
2017	2840 (10.5%)	133 (7.5%)	2707 (10.7%)	
2018	3744 (13.8%)	181 (10.2%)	3563 (14.1%)	
2019	3884 (14.3%)	287 (16.2%)	3597 (14.2%)	
2020	3601 (13.3%)	278 (15.7%)	3323 (13.1%)	
2021	4517 (16.7%)	292 (16.5%)	4225 (16.7%)	
2022	4544 (16.8%)	337 (19.0%)	4207 (16.6%)	
2023	3946 (14.6%)	261 (14.7%)	3685 (14.5%)	
Hernia Recurrence, 30-day Follow-up				0.7538
Yes	41 (0.2%)	3 (0.2%)	38 (0.2%)	
No	22311 (99.8%)	1496 (99.8%)	20815 (99.8%)	
Hernia Recurrence at 1-year on PRO/Surgeon Report				<0.0001
Yes	539 (6.8%)	75 (11.0%)	464 (6.4%)	
No	7414 (93.2%)	607 (89.0%)	6807 (93.6%)	
Surgical Site Occurrences, 30-day Follow-up				<0.0001
Yes	1128 (5.0%)	33 (2.2%)	1095 (5.3%)	
No	21224 (95.0%)	1466 (97.8%)	19758 (94.7%)	
Surgical Site Infection, 30-day Follow-up				0.1842
Yes	39 (0.2%)	5 (0.3%)	34 (0.2%)	
No	22313 (99.8%)	1494 (99.7%)	20819 (99.8%)	
Readmission, 30-day Follow-up				0.0459
Yes	183 (0.8%)	19 (1.3%)	164 (0.8%)	
No	22169 (99.2%)	1480 (98.7%)	20689 (99.2%)	

**Fig. 1:**
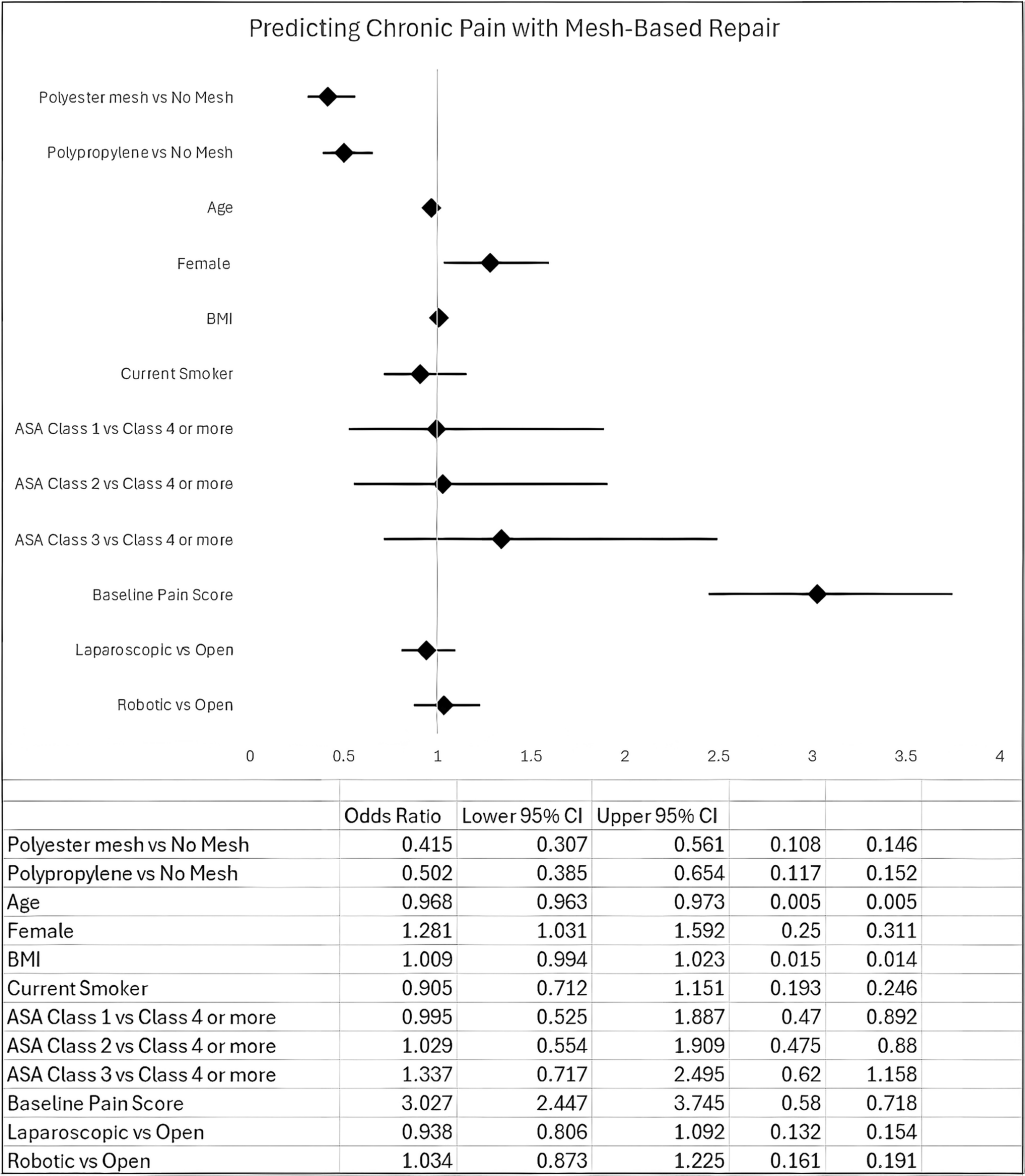
Chronic Pain (≥6-month) for Polyester and Polypropylene Mesh Repair versus No-Mesh Repair

On logistic regression analysis, both polyester (OR 0.4, CI 0.28–0.69) and polypropylene (OR 0.4, CI 0.27–0.57) mesh were protective against hernia recurrence and had similar SSO for polyester (OR 1.9, CI 1.01–3.51) and polypropylene (OR 1.9, CI 1.06–3.51) mesh repair compared to no-mesh repair. Upon adjusting for patient characteristics, baseline pain, and surgical approach, both polyester (OR 0.4, CI 0.31–0.56) and polypropylene (OR 0.5, CI 0.39–0.65) mesh had lower ≥6-month EuraHS-QoL pain domain score compared to no-mesh repair, suggesting mesh repair to be protective against chronic pain [Fig. 1]. Female patients (OR 1.2, CI 1.03–1.6) and patients with higher baseline pain scores (OR 3.0, CI 2.45–3.75) had higher risk for chronic pain. However, current smoking status, BMI, and age had no statistically significant impact on chronic pain outcome for mesh versus no-mesh IHR.

**Fig. 2:**
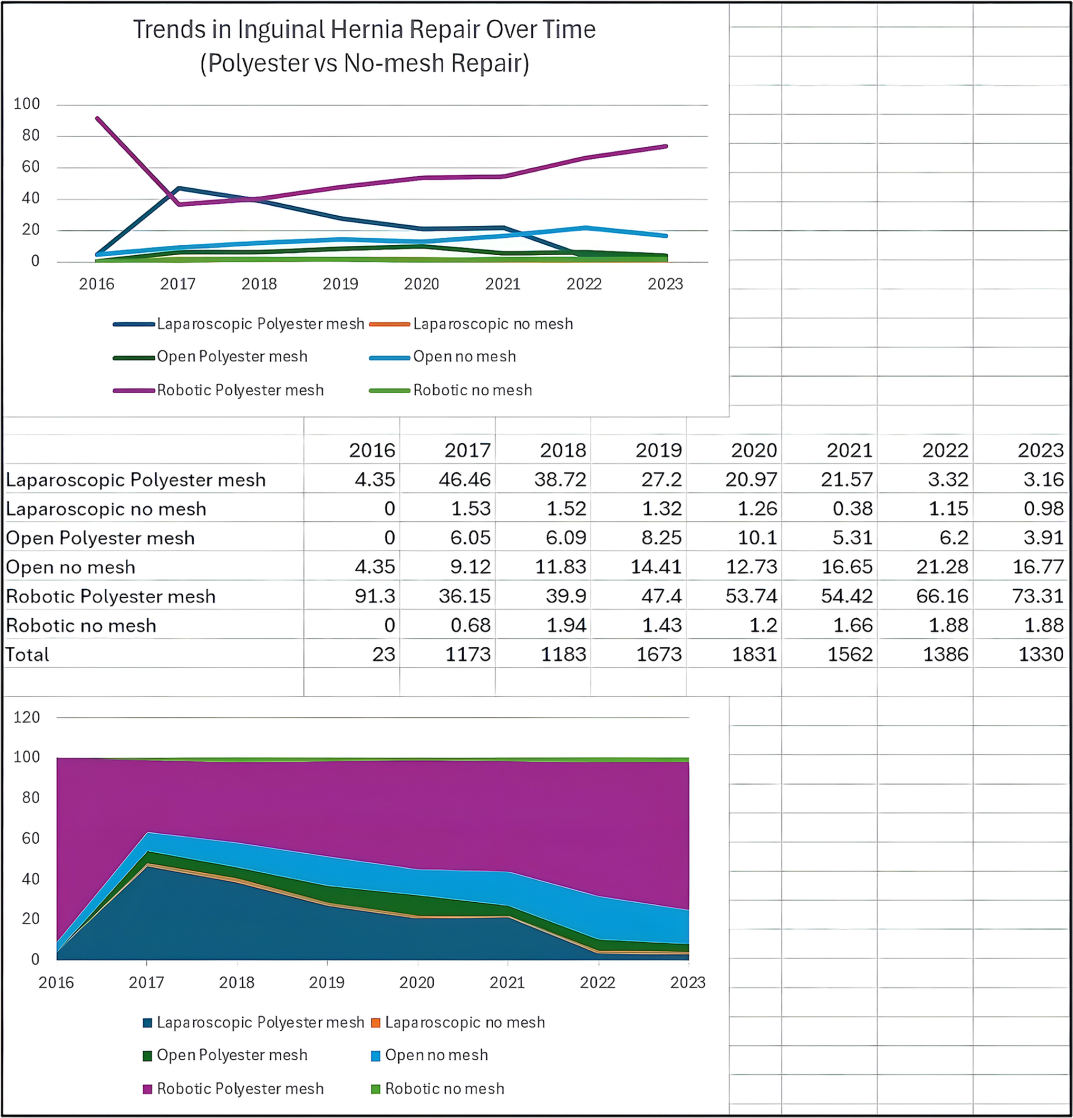
Trends in Inguinal Hernia Repair (Polyester versus No-mesh Repair)

**Fig. 3:**
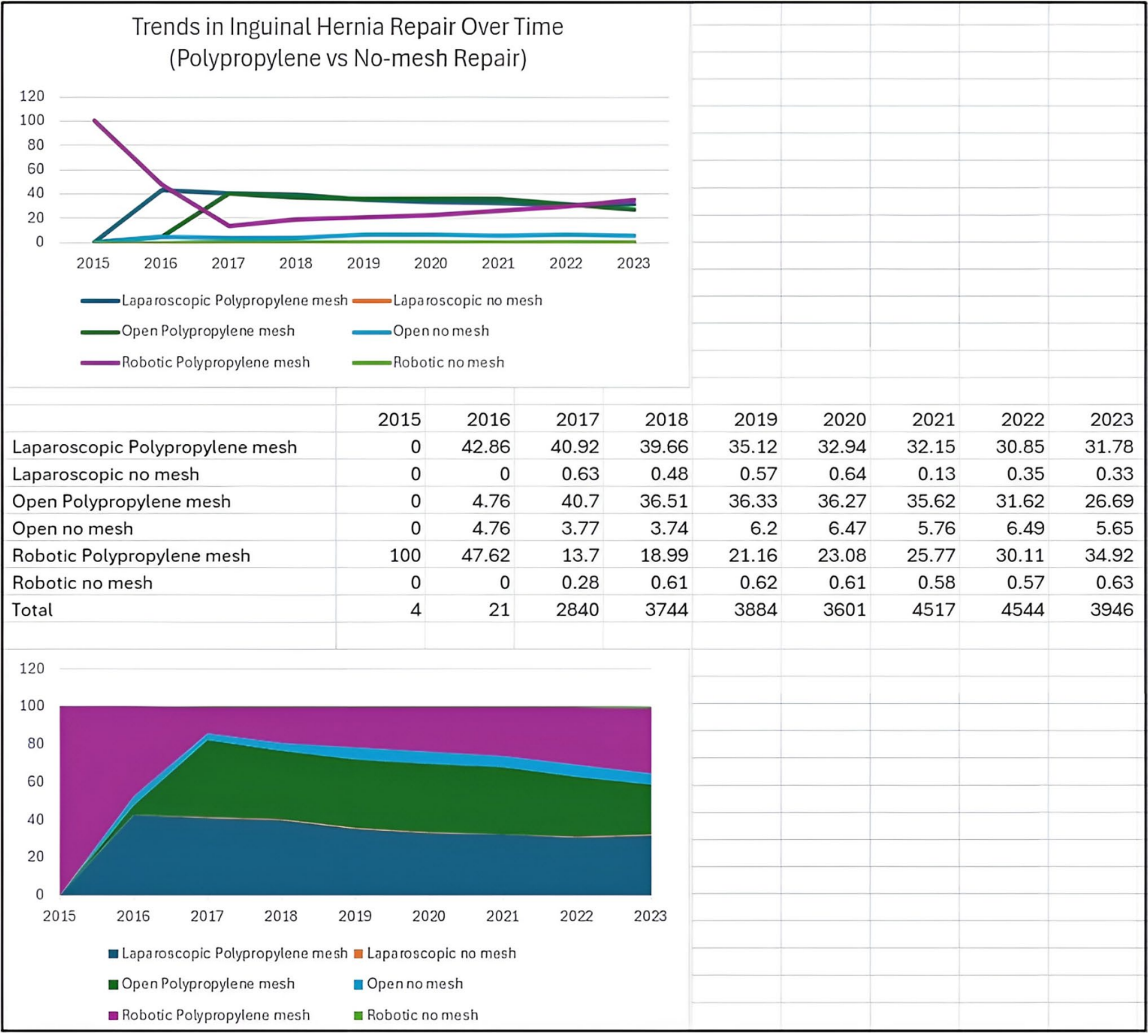
Trends in Inguinal Hernia Repair (Polypropylene versus No-mesh Repair)

In adjusted analyses evaluating mesh type and operative approach simultaneously, both polyester and polypropylene mesh were independently associated with lower odds of chronic postoperative pain compared with no-mesh repair. In contrast, operative approach (open, laparoscopic, or robotic) was not independently associated with chronic pain outcomes. Female sex and higher baseline pain scores remained significant predictors of chronic pain. Adjusted associations between mesh type, operative approach, and chronic postoperative pain are shown in Supplementary Table S2.There were no statistically significant differences in these clinical outcomes for polyester versus polypropylene mesh usage based on different surgical approaches for IHR. However, over the last decade, there has been a steady increase in robotic IHR and this temporal trend coincided with greater utilization of polyester mesh for robotic repairs [Fig. 2]. The use of polypropylene mesh for IHR was found to have an overall steady utilization trend irrespective of the surgical approach [Fig. 3].

## Discussion

This study attempts to bridge the knowledge gap in understanding the clinical outcomes of polyester and polypropylene mesh used in IHR utilizing a national database which could be vital for one of the most common surgical procedures in the United States.

### Polyester and polypropylene mesh inguinal hernia repair

Our multicenter national database-based study found no significant difference between polyester and polypropylene mesh IHR for SSO, hernia recurrence, or chronic pain, irrespective of open, laparoscopic, or robotic IHR approach. This suggests that the choice of mesh material, while important in terms of its biological properties, may not be the sole determinant of clinical outcomes for IHR, as previously thought. A surgeon’s mesh selection for IHR could be based on patient characteristics, resource availability, cost, individual experience, and or practice preference [15, 17]. Our study results support mesh selection based on these factors over solely mesh property-based selection for IHR.

In this large cohort of patients who underwent elective, initial IHR, both polyester and polypropylene mesh repairs demonstrated lower recurrence rates compared to no-mesh repairs at the 1-year follow-up. This aligns with the well-established benefits of mesh-based repair in decreasing the risk of hernia recurrence and the overall decrease in the trend for no-mesh elective IHR [8, 18]. The higher absolute 1-year recurrence rates observed in this study likely reflect differences in outcome definition and follow-up capture, as recurrence was defined using a composite registry variable incorporating both patient- and surgeon-reported events; importantly, relative comparisons within the dataset consistently demonstrate a protective effect of mesh-based repair. Although recurrence rates observed in this study appear higher than those reported in randomized trials, these values reflect real-world outcomes from a national registry encompassing a heterogeneous patient population. Despite this variability, mesh-based repair consistently demonstrated a lower risk of recurrence compared to no-mesh repair, reinforcing its established benefit in elective IHR.

In the immediate post-operative period, both polyester and polypropylene mesh repairs were associated with a higher incidence of SSO compared to no-mesh repairs. This incidence rate of SSO for mesh repair is similar to the previously reported data for SSO for IHR with mesh [18]. However, there was no statistical difference in SSO on logistic regression between these two widely used mesh types. Though the outcome for SSO is relatively higher in mesh versus no-mesh IHR, this could be multifactorial and not solely based on the mesh type.

### Mesh usage and chronic pain

Chronic pain after mesh-based IHR is one of the most feared complications, affecting approximately 11% of patients and resulting in lifestyle limitations for almost one-third of the patients undergoing mesh-based IHR [5]. However, the literature evaluating chronic pain after IHR is limited by either duration of postoperative follow-up to only 3 months or surgical technique with more studies on open mesh-based IHR [5, 19]. Per a patient-reported outcomes study conducted in Sweden on patients who underwent open mesh-based IHR, mesh type in itself was not associated with chronic pain at 1-year follow-up from surgery as opposed to gender and age [20]. The current literature highlights the variability in chronic pain outcomes deterring its generalization. Our study utilized the EuraHS-QoL pain domain scores from a validated national database of ACHQC. Our study demonstrates the protective nature of mesh-based IHR for chronic pain irrespective of mesh type compared to no-mesh repair which contradicts previously reported data. In adjusted analyses accounting for mesh type and operative approach, surgical technique was not independently associated with chronic pain, whereas both polyester and polypropylene mesh were associated with lower chronic pain risk compared to no-mesh repair. Our study also demonstrated that female patients and patients with high baseline pain scores had a higher risk for chronic pain from mesh-based IHR. This highlights that chronic pain after IHR might be multifactorial, independent of the mesh type. Future prospective and patient reported outcomes studies would prove beneficial to validate our results.

### Robotic inguinal hernia repair

Robotic surgery has continued to be adapted for a broad range of common surgical procedures, with IHR being no exception [21]. Mesh repair is the standard of care for elective IHR, irrespective of the surgical approach [3, 4]. Our study demonstrates a progressive increase in the trend for robotic approach in IHR over the last decade and a decrease in no-mesh repair around the same time. There was a preferential increase in polyester mesh usage without affecting the current prevalence of polypropylene mesh usage in IHR. This could be because of the perceived advantages in mesh handling and resistance to stretching affecting surgeon preference [6, 7, 13]. The increase in the trend for polyester mesh in robotic IHR could be temporal with the invention of anatomically contoured and self-adhesive polyester meshes and increased adaptation for robotic surgery [15, 17, 21]. However, surgeon rationale and decision making cannot be determined from registry data.

## Limitations

In addition to the inability to assess surgeon rationale from registry data, this study has the inherent limitations of a retrospective registry design with selection bias, lack of randomization and missing data. Additionally, recurrence events were captured through registry-based follow-up rather than standardized diagnostic confirmation, which may influence absolute recurrence estimates. This could limit generalization across all patient populations. Though large sample size owing to ACHQC database is the strength of this study, this limited our ability to evaluate more granular data that could have an impact on the overall outcome. Though this study attempts to capture ten-year clinical outcomes of polyester versus polypropylene mesh-based IHR, the ACHQC data is most robust from 2017 making the results more accurate for the past seven years instead. Mesh density, an additional mesh characteristic evaluated in prior studies, is not recorded in ACHQC and could not be included in this analysis. Future work incorporating density and other mesh structural properties may clarify their relationship to recurrence and chronic pain. In spite of these limitations, this study provides valuable insight into the clinical outcomes for mesh-based open and minimally invasive IHR.

## Conclusion

Irrespective of the surgical approach and mesh type, there was no difference in the 30-day SSO or 1-year hernia recurrence in IHR. Contrary to generalized belief, mesh-based repair and posterior approach may be protective against chronic postoperative pain. These findings suggest that the choice of mesh material may be less critical to patient outcomes than previously believed. Factors such as surgical technique, surgeon experience, and resource utilization play a larger role in shaping clinical decisions and outcomes. Our study calls attention to the need for prospective studies and to focus on surgeon preferences and resource utilization that could impact practice guidelines.

## Data Availability

The data that support findings of this study were derived from the ACHQC database. De-identified clinical data utilized in this study are available from ACHQC upon request and following appropriate institutional approval.

## Supporting Information

**ESM 1.**

**ESM 2.**

## Abbreviations

ACHQC: Abdominal Core Health Quality Collaborative
ASA Classification: American Society of Anesthesiologists Classification
BMI: Body Mass Index
CI: Confidence Interval
IHR: Inguinal Hernia Repair
IQR: Interquartile Range
aOR: Adjusted Odds Ratio
*P*-Value: Probability Value
SAS: Statistical Analysis System
