# Supplementary material for: Does the Type of Permanent Mesh Matter for Inguinal Hernia Repair?
Source: Hernia. 2026 Apr 28;30(1):185. doi: 10.1007/s10029-026-03615-9 (PMC13124928; doi:10.1007/s10029-026-03615-9)
Supplement: Supplementary file 1 — ESM 1. [file 10029_2026_3615_MOESM1_ESM.docx]

## Supplementary Table S1. One-year hernia recurrence by mesh type

| Mesh type | Total patients (n) | Hernia recurrence at 1 year, n (%) | p-value |
| --- | --- | --- | --- |
| No mesh | 1,770 | 75 (4.24%) | — |
| Polyester mesh | 8,391 | 156 (1.86%) | <0.0001 |

Footnote: One-year hernia recurrence was defined using the Abdominal Core Health Quality Collaborative (ACHQC) composite recurrence variable, which incorporates both patient-reported and surgeon-reported recurrence within one year. P-value derived from chi-square testing.
