# Supplementary material for: Does the Type of Permanent Mesh Matter for Inguinal Hernia Repair?
Source: Hernia. 2026 Apr 28;30(1):185. doi: 10.1007/s10029-026-03615-9 (PMC13124928; doi:10.1007/s10029-026-03615-9)
Supplement: Supplementary file 2 — ESM 2. [file 10029_2026_3615_MOESM2_ESM.docx]

## Supplementary Table S2. Adjusted odds of chronic postoperative pain by mesh type and operative approach

| Variable | Adjusted Odds Ratio | 95% Confidence Interval | p-value |
| --- | --- | --- | --- |
| Polyester mesh vs no mesh | 0.42 | 0.31–0.56 | <0.001 |
| Polypropylene mesh vs no mesh | 0.50 | 0.39–0.65 | <0.001 |
| Laparoscopic vs open | 0.94 | 0.81–1.09 | 0.41 |
| Robotic vs open | 1.03 | 0.87–1.23 | 0.70 |
| Female vs male | 1.28 | 1.03–1.59 | 0.03 |
| Baseline pain present vs absent | 3.03 | 2.45–3.75 | <0.001 |

Footnote: Adjusted odds ratios derived from multivariable penalized logistic regression including mesh type, operative approach, age, sex, BMI, smoking status, ASA class, and baseline pain. Chronic pain defined as pain persisting ≥6 months postoperatively based on EuraHS-QoL pain domain.
